# Multimodality magnetic resonance imaging for the diagnosis of high-flow priapism following a straddle injury: A case report

**DOI:** 10.1097/MD.0000000000022618

**Published:** 2020-10-09

**Authors:** Ping Zhu, Shufeng Fan, Junyi Xiang

**Affiliations:** Department of Radiology, The Second Affiliated Hospital of Zhejiang Chinese Medical University, Hang Zhou, Zhejiang, China.

**Keywords:** diagnosis, high-flow priapism, multimodality magnetic resonance imaging, straddle injury

## Abstract

**Rationale:**

Priapism is a common urologic emergency, but high-flow penile priapism (HFP) caused by trauma is very rare. Therefore, HFP diagnosis and treatment are still not standardized.

**Patient concerns:**

A 29-year-old man was admitted to the urology department of our hospital on August 01, 2019, due to “persistent penile erection caused by a straddle injury.”

**Diagnosis:**

On July 17, 2019, the patient underwent Doppler ultrasonography, which indicated swollen corpus cavernosum.

**Interventions:**

The patient took over-the-counter anti-inflammatory drugs but the erectile state of the penis remained unchanged. A second perineal injury resulted in hospital admission. Multimodality magnetic resonance imaging (MRI) scan showed nodular abnormal signals at the right corpus cavernosum root. Subsequently, selective arterial interventional angiography confirmed the MRI findings. Spring coils were then inserted for embolization, and the pseudoaneurysm, fistula, and priapism disappeared.

**Outcomes:**

Two months after surgery, sexual stimuli could normally cause penile erection, with normal hardness. The patient's sexual life returned to normal 3 months after surgery.

**Conclusion:**

Multimodality MRI is very effective in detecting high blood flow priapism. Its application would improve the clinical management of this ailment.

## 1 Introduction

Priapism refers to a state of continuous penile erection exceeding 4 hours, independent of sexual desire or stimulation, with an incidence approximating 1.5/100,000. It comprises the low-flow (ischemic, painful) and high-flow (nonischemic, painless) types.^[1]^ Priapism represents a common urologic emergency, but high-flow penile priapism (HFP) caused by trauma is very rare (about 15% of all priapism cases) in clinic.^[2,3]^ Therefore, HFP diagnosis and treatment are still not standardized. Multimodality magnetic resonance imaging (MRI) as an important examination method is noninvasive and easy to operate, and could directly display the pseudoaneurysm and penile artery-cavernous fistula caused by penile artery damage. In this study, a patient with post-traumatic HFP was reported. Multimodality MRI [T1-weighted image (T1WI), FS-T2WI, diffusion-weighted imaging (DWI), apparent diffusion coefficient (ADC), and enhanced scan] was used for diagnosis, which was confirmed by selective arterial interventional radiography.

## 2 Case report

A physically healthy 29-year-old male patient with no history of related functional diseases was admitted to the urology department of our hospital on August 01, 2019, due to “persistent penile erection caused by a straddle injury for more than 1 month and aggravation for 1 day.” Perineal numbness was felt immediately after the straddle injury, and perineal pain occurred 10 minutes later. The perineum and penis swelled gradually, accompanied by persistent penile erection without sexual stimulation, with a hardness of about grade 2 according to the Erection Hardness Grading Scale (EHGS). EHGS scores range from 0 (penis not enlarged) to 4 (penis completely hard and fully rigid).^[4]^ There was no obvious improvement or aggravation before and after urination. The patient also had local congestion, but no open bleeding, dysuria, blood in the urine, painful urination, or fever. On July 17, 2019, the patient underwent Doppler ultrasonography at a local hospital, which indicated swollen corpus cavernosum, but a clear diagnosis could not be reached. The patient did not pay much attention and took over-the-counter anti-inflammatory drugs (specific information unknown), and perineal pain and congestion gradually improved. However, the erectile state of the penis remained unchanged, and there was pain after pressing, with no significant improvement. The preceding day, the patient had another straddle injury at work, and erection hardness was increased, resulting in hospital admission. At admission, examination showed normal penile development; the penis was in an erectile state, with a hardness of about grade 2. The middle part of the penis was dorsally curved, with pain upon pressing. The skin color of bilateral scrotum was normal, and the testicles and epididymides were normal. Routine urine test showed 8 erythrocytes and 28 leucocytes in urine, with no obvious abnormality in blood routine. Blood gas analysis showed a pH of 7.445, PCO_2_ at 34.3 mm Hg, PO_2_ at 145.0 mm Hg, and SO_2_ at 99.3%, indicating that the penis was in a high-flow state.

**Figure 1:**
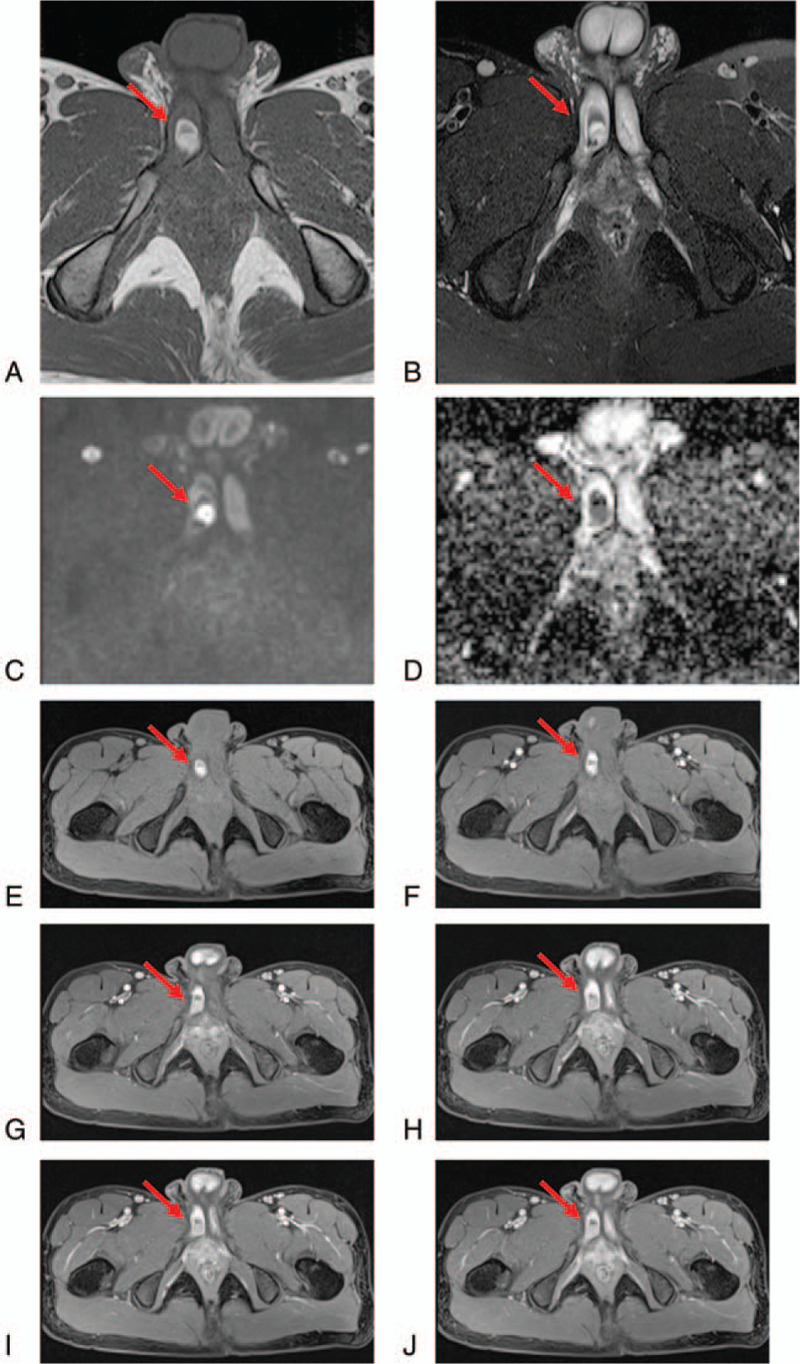
Nodular abnormal signal shadows at the right corpus cavernosum root. A, T1WI dominated by high signals, accompanied by internal low-signal shadows. B, FS-T2WI showing uneven circular nodular high signals, surrounded by low-signal shadows. C, Diffusion-weighted imaging (DWI) showing obvious high signals. D, Apparent diffusion coefficient (ADC) suggesting low signals. E–J, Contrast-enhanced scanning showing progressive and significant enhancement inside and around the above abnormal signals, with the right corpus cavernosum displayed with continuous enhancement.

Upon admission, multimodality MRI scan showed nodular abnormal signals at the right corpus cavernosum root. T1WI was dominated by high signals, accompanied by internal low-signal shadows. FS-T2WI showed uneven circular nodular high signals, surrounded by low-signal shadows. The DWI revealed obvious high signals, and the ADC values indicated low signals, lesion size approximating 1.2 × 1.8 cm. Enhanced scanning showed significant enhancement within and around the above abnormal signals. The right corpus cavernosum was readily displayed, with continuous enhancement. The right and dorsal penile arteries were slightly thickened, and the left penile artery and penile corpus cavernosum showed no abnormal contrast enhancement. In addition, penile shape and size showed no overt abnormalities. Subacute hematoma of penile artery pseudoaneurysm at the right corpus cavernosum root was diagnosed, with penile artery-cavernous fistula (Fig. 1).

**Figure 2:**
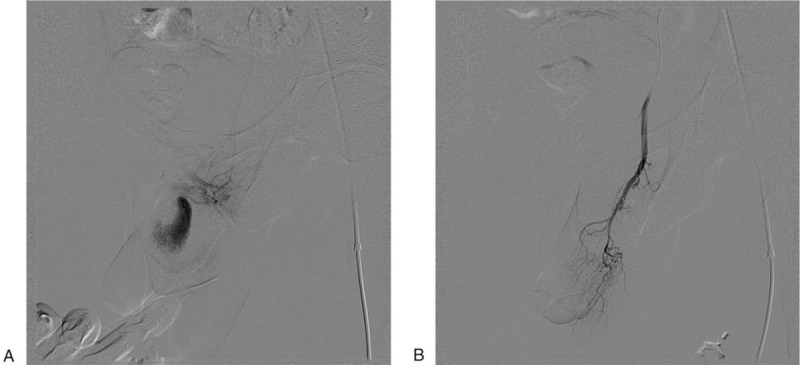
Right perineal internal arteriography. A, Pseudoaneurysm of the penile artery and cavernous fistula, with the corpus cavernosum displayed in the arterial phase. B, A spring ring was inserted for embolism, and the pseudoaneurysm and fistula disappeared after successful treatment.

Subsequently, the patient underwent selective arterial interventional angiography, revealing right penile artery pseudoaneurysm, with a diameter of about 2.0 cm, as well as thrombus generation and cavernous fistula formation, which confirmed the diagnosis achieved by MRI. Two 2.0 × 5.0 mm and one 3.0 × 2.5 mm spring coils (Boston Scientific Corporation: 300 Boston Scientific Way, Marlborough) were then inserted for embolization. After successful embolization, the pseudoaneurysm and fistula disappeared (Fig. 2). Priapism also disappeared, and erection hardness changed from continuous grade 2 to grade 0. Two months after surgery, sexual stimuli could normally cause penile erection, whose hardness was normal (grade 4). The patient's sexual life returned to normal 3 months after surgery. The institutional review board of our hospital approved the study. Written informed consent was obtained from the patient for publication of this case report and accompanying images.

## 3 Discussion

Priapism is a rare pathological erectile state, which could occur at any age, including newborns. Children aged 5 to 10 years and adults aged 20 to 50 years are the age groups most affected.^[5]^

Compared with low-flow priapism, high-flow priapism is rarer and mostly caused by trauma. After cavernous artery injury, arterial blood flows directly from the injured site to the sinus in the corpus cavernosum without returning to the vein through the spiral artery, forming a persistent high-flow state. Other causes include hereditary metabolic disorders, hematological diseases, local vascular malformations and spontaneous rupture of angiomas.^[6]^ The current case was caused by trauma.

Currently, the diagnosis of high-flow priapism is mainly based on medical history, physical examination, laboratory examination (eg, blood gas analysis of the corpus cavernosum), and auxiliary examination such as color Doppler ultrasound and angiography. Most patients with high-flow priapism have a history of perineal trauma. Physical examination shows incomplete erection of the penis, which could be completely erected if stimulated. Blood gas analysis of the corpus cavernosum is an effective method for diagnosing this disease.^[7]^ Because blood in the corpus cavernosum originates from the ruptured internal perineal artery or the cavernous artery, it is bright red. Blood gas indexes are close to arterial blood levels, with increased pathological arterial blood flow. Color Doppler ultrasound in patients with carotid-cavernous fistula could show colored blood flow signals, with the corpus cavernosum revealing arterial spectrum. Chiou et al^[8]^ proposed that color Doppler ultrasound is useful for assessing relief in arteriogenesis and veno-occlusion and making decision for subsequent therapy.

To date, multimodality MRI has not been performed to diagnose traumatic priapism.^[9]^ MRI mainly depends on tissue proton mass and movement in the magnetic field. After trauma, the cavernous artery is injured, and blood accumulates in the sinus of the corpus cavernosum. Cases with arterial-cavernous fistula could also form pseudoaneurysms. The MRI sequence could clearly show the complex pathological changes and blood hemoglobin modifications after bleeding: oxygenated hemoglobin → deoxyhemoglobin → methemoglobin → hemosiderin.^[10]^ Changes in signal intensity of the lesion could also be observed. The present patient had a hematoma and pseudoaneurysm after trauma, and was in the transition from deoxyhemoglobin to methemoglobin at the time of admission, which reflects the middle and late stages of subacute hemorrhage. Therefore, T1WI was dominated by high signals, accompanied by internal low-signal shadows, whereas FS-T2WI showed uneven circular nodular high signals, surrounded by low-signal shadows; DWI showed obvious high signals, while ADC values indicated low signals. Contrast-enhanced MRI showed significant enhancement inside and around the above abnormal signals. Because the right penile artery was injured, the corpus cavernosum was displayed with continuous enhancement. In pediatric HFP, it was suggested that an observation period should be introduced in the management algorithm of HFP, to avoid unnecessary surgical intervention.^[11]^

In conclusion, compared with traditional color Doppler ultrasound and selective arterial interventional angiography, multimodality MRI is more effective in detecting high blood flow priapism. Indeed, it is noninvasive and simple to operate, and allows visualization of pseudoaneurysms and penile arterial-cavernous fistula caused by penile arterial injury, accurately reflecting the pathological distribution of blood and clearly diagnosing high blood-flow priapism. Therefore, multimodality MRI is expected to become a routine examination technique with improved clinical diagnosis of high blood-flow priapism. Its application would improve the clinical management of this ailment.

## Acknowledgments

The authors thank the current patient for willingness to share the clinical data.

## Author contributions

**Conceptualization:** Shufeng Fan.

**Investigation:** Ping Zhu, Junyi Xiang.

**Methodology:** Ping Zhu, Junyi Xiang.

**Writing – original draft:** Ping Zhu.

**Writing – review & editing:** Shufeng Fan.

## Abbreviations

Abbreviations: ADC = apparent diffusion coefficient, DWI = diffusion-weighted imaging, EHGS = Erection Hardness Grading Scale, HFP = high-flow penile priapism, MRI = magnetic resonance imaging, T1WI = T1-weighted image.
